# Editorial: The Role of Working Memory and Executive Function in Communication under Adverse Conditions

**DOI:** 10.3389/fpsyg.2016.00148

**Published:** 2016-02-11

**Authors:** Mary Rudner, Carine Signoret

**Affiliations:** Department of Behavioural Sciences and Learning, Linnaeus Centre HEAD, Swedish Institute for Disability Research, Linköping UniversityLinköping, Sweden

**Keywords:** cognition, communication, adverse conditions, hearing, deafness

Communication is fundamental for social participation with communication difficulties often leading to social isolation and depression. Nevertheless, everyday communication is often hindered either by internal factors such as sensory loss, or by external factors including the background noise that commonly occurs in places where people meet, such as restaurants, schools, and railway stations. In such adverse conditions, working memory and executive functions have been proposed to play a critical role in communication. Thus, the role of cognition in hearing is a central theme in the field of Cognitive Hearing Science and has crystalized as one of the main themes of this research topic. This is reflected in papers reporting the role of cognition in hearing in persons with varying sensory and cognitive status and varying degrees of language knowledge, over the lifespan. Another theme represented in this topic is rehabilitation in the form of amplification and training. Importantly, the broad remit of the research topic is reflected in papers addressing cognition and communication in children with sensory and cognitive issues as well as adults and children who are profoundly deaf and use sign language to communicate. Apart from the impressive number of empirical studies, there are several theoretical contributions to the field.

The observation of consistent correlations between cognitive skills and the ability to understand speech under adverse conditions has played an important role in driving the field of Cognitive Hearing Science. In particular, it has been reported repeatedly that working memory explains variance in the ability to recognize speech in noise above and beyond differences in hearing thresholds. In the current research topic, Heinrich et al. report a study showing, in line with previous work, that individual differences in sensory and cognitive skills explain variance in the ability of older listeners with mild sensorineural hearing loss to process speech. However, they also show that the relative explanatory value of these skills depends on the linguistic demands of the particular speech test, with hearing sensitivity being more important at the phoneme level and cognition at the sentence level. Further, they reported associations between self-reported aspects of auditory functioning and speech intelligibility. Smith and Pichora-Fuller compared performance on the reading span test (RS), which is a well-established measure of working memory delivered visually, and the Word Auditory Recognition and Recall Measure (WARRM), a newer measure of working memory with auditory delivery, which they propose is more ecologically valid. WARRM performance was better and more varied than RS performance in all groups tested (young adults with normal hearing, young-older hard-of-hearing adults and old-older hard-of-hearing adults) and the authors suggested that this pattern of performance indicates WARRM may be a useful clinical test. However, no consistent pattern of correlations was found between the two cognitive measures and measures of the ability to understand speech in noise. Smith and Pichora-Fuller suggest that there is a need for a more consistent approach to determine in more ecologically relevant conditions associations between working memory and speech understanding. During speech comprehension, encoding of new memories may be hampered by interference from established memories; this is known as proactive interference. Ellis and Rönnberg studied whether the ability to suppress such interference was associated with speech recognition in noise in older hard-of-hearing adults. In line with previous work on individuals with normal hearing, they did find an association, but only when hearing was unaided. They suggested that the cognitive flexibility reflected by performance on their cognitive task is a key factor in listening ability.

Experimental approaches are adopted by another set of studies studying the role of cognition in communication. Kidd and Humes used an auditory working memory task to determine differences in the ability of older and younger listeners to keep track of who said what. They found that although older listeners were slower, they were almost as accurate as younger listeners. However, older listeners did not benefit from consistent mapping of target speaker and location in the same way as younger listeners. Doherty and Desjardins investigated how amplification influenced auditory working memory performance in hard-of-hearing listeners who were fitted with hearing aids for the first time. They found that amplification improved working memory and the overall pattern of results suggested that this was due to perceptual benefit rather than cognitive change. Moradi et al. used a gating paradigm to determine whether background noise influences how much of the auditory signal is needed before identification of its linguistic content is achieved. Results showed that more auditory signal was required in noise and that this effect was modulated by both working memory and executive function. DiDonato and Surprenant investigated how speech manner influences the ability of older and younger listeners to remember auditory information with ecological relevance. They found that older listeners could remember medical information better when it was presented clearly rather than conversationally, even in background noise. The electrophysiology study by Petersen et al. investigated how working memory indexed by neural oscillations in a low frequency (alpha) band, is influenced by increasing stimulus degradation and working memory load, in hard-of-hearing individuals. In line with previous work in individuals with normal hearing thresholds, performance decreased and alpha power increased with greater stimulus degradation and working memory load. However, at the highest levels of degradation and working memory load, alpha power dropped for the participants with the greatest degree of hearing loss, suggesting a breakdown in an important neural mechanism that may support listening in noise.

If cognitive resources are consumed during listening in noise as indicated by the association between working memory and listening performance, fewer resources, or less cognitive spare capacity (CSC) will be available for higher level processing of the message. The research topic includes a set of studies investigating this phenomenon. In line with previous work, Keidser et al. found that performance on the CSC Test (CSCT) was influenced by some of the manipulated parameters (but not seeing the talker's face) and that there was no consistent relation between CSCT and RS. Further, there was no relation between CSCT and a novel speech comprehension test presented in noise. Using the Auditory Inference Span Test (AIST), a sentence-based test which involves storage and processing of the message, Rönnberg et al. showed that, even when audibility is relatively well-maintained, processing of a spoken message becomes harder for listeners with normal hearing thresholds when noise level increases, but only when the noise is speech-like. This suggests that speech-like background reduces CSC. Lin and Carlile used a version of AIST to investigate the listening costs associated with shifts in spatial attention during conversational turn-taking in listeners with normal hearing thresholds. They found that listening costs were dependent on load and cognitive complexity but not on the nature of the spatial shift.

Hearing aid signal processing is designed to improve speech understanding. It is important to determine whether this is actually the case and at the same time identify any contingent cost in terms of cognitive function. In this research topic, Souza et al. investigated the role of working memory in speech intelligibility in noise with hearing aid signal processing. The data corroborated previous results showing that individuals with low working memory capacity may benefit more from signal processing that better retains the signal envelope. Neher studied whether working memory and executive function were related to speech recognition in noise performance with hearing aid signal processing as well as preference for different hearing aid fittings in older hearing aid users. His study found that working memory was related to performance with directional microphones while executive function was related to preference for noise reduction.

Ferguson and Henshaw reviewed three auditory training studies and conclude that training which combines auditory and cognitive demands is most likely to benefit hard-of-hearing adults in real-world listening situations. Henshaw et al. argue that training benefit is dependent on uptake, engagement and adherence. Their study showed that uptake was associated with extrinsic motivation (e.g., hearing difficulties) while engagement and adherence were influenced by both intrinsic (e.g., a desire to achieve higher scores), and extrinsic (e.g., to help others with hearing loss) motivations.

An atypical language model can lead to particular involvement of working memory and executive function in language processing. Kilman et al. studied the amount of disturbance perceived by hard-of-hearing listeners and listeners with normal hearing thresholds when attending to a target talker against a multitalker background. Speech was either native or non-native. Results showed that hard-of-hearing participants were particularly disturbed by native speech masked by native babble. Hygge et al. investigated how nativeness of speech influenced the ability to recognize and recall speech in different levels of background noise. They found that recall was more sensitive than recognition to both factors and thus a better indicator for the acoustics of learning.

Because profoundly deaf individuals do not have access to sound, reading and other academic skills may develop differently from those of individuals with normal hearing. Hirshorn et al. assessed the impact of language experience on predictors of reading comprehension in deaf readers. They found that while English phonological knowledge best predicted reading comprehension in oral deaf individuals, free recall was a better predictor in deaf native signers. Marshall et al. investigated the relationship between working memory and language in deaf signing children who were either native or non-native users of British Sign Language compared to hearing children. The non-native signers performed less accurately than both the native signers and the hearing children. Further, vocabulary predicted working memory, suggesting that the good language skills resulting from early acquisition are important for development of working memory.

A number of the papers in the research topic report studies investigating cognitive aspects of language development in children with disabilities. In a perspective article, Sandgren and Holmström discuss the clinical challenge of assessing language impairment in bilingual children and present work suggesting that measuring executive function may be a useful approach. In a mini-review Lyberg-Åhlander et al. discuss their recent work investigating how children's listening comprehension is influenced by speaker voice quality and background noise, as well as the child's own cognitive capacity. They highlight risk of underachievement when speech is delivered in a dysphonic (hoarse) voice, especially when the task is simple or the child's capacity is stretched. In another mini-review, Sandgren et al. summarize their work on referential communication showing that while children with sensorineural hearing impairment are active and competent conversational partners, their conversational strategies are distinct from those of their peers with normal hearing, even when the listening situation is optimized.

Finally, two perspective articles round off the research topic. Lemke and Scherpiet discuss communication from an aging perspective, and the psycho-social impact of sensory and cognitive decline. Wingfield et al. discuss the Ease of Language Understanding (ELU) model (Rönnberg et al.) as one of the few attempts to offer a fully encompassing framework for language understanding. They identify its strengths and point out avenues for future work.

Altogether, the articles in this research topic demonstrate the crucial role of cognition, including working memory and executive functions but also cognitive flexibility and cognitive load, in communication under adverse conditions, in different modalities, and over the lifespan.

## Author contributions

MR prepared the first draft of the editorial, and CS and MR contributed equally to completion of the final version.

## Funding

This work was supported by funding from the Swedish Research Council to the Linnaeus Centre HEAD, Swedish Institute for Disability Research, Department of Behavioral Sciences and Learning, Linköping University.

### Conflict of interest statement

The authors declare that the research was conducted in the absence of any commercial or financial relationships that could be construed as a potential conflict of interest.

